# Approximate quantum circuit compilation for proton-transfer kinetics on quantum processors

**DOI:** 10.1039/d5cp04097c

**Published:** 2026-01-14

**Authors:** Arseny Kovyrshin, Dilhan Manawadu, Edoardo Altamura, George Pennington, Benjamin Jaderberg, Sebastian Brandhofer, Anton Nykänen, Aaron Miller, Walter Talarico, Stefan Knecht, Fabijan Pavošević, Alberto Baiardi, Francesco Tacchino, Ivano Tavernelli, Stefano Mensa, Jason Crain, Lars Tornberg, Anders Broo

**Affiliations:** a Predictive Science, Digital and Automation, Pharmaceutical Sciences, R&D, AstraZeneca Gothenburg Pepparedsleden 1 Molndal SE-431 83 Sweden arseny.kovyrshin@astrazeneca.com; b Department of Microtechnology and Nanoscience, Chalmers University of Technology Gothenburg SE-412 96 Sweden; c The Hartree Centre, STFC, Sci-Tech Daresbury Warrington WA4 4AD UK; d Yusuf Hamied Department of Chemistry, University of Cambridge Lensfield Road Cambridge CB2 1EW UK; e IBM Quantum, IBM Research Europe, Hursley Winchester SO21 2JN UK; f IBM Quantum, IBM Germany Research & Development Böblingen Germany; g Algorithmiq Ltd., Kanavakatu 3C FI-00160 Helsinki Finland; h School of Physics, Trinity College Dublin, College Green Dublin 2 Ireland; i QTF Centre of Excellence, Department of Physics, University of Helsinki P.O. Box 43 FI-00014 Helsinki Finland; j IBM Quantum, IBM Research Zürich 8803 Rüschlikon Switzerland; k IBM Research Europe, The Hartree Centre, STFC, Sci-Tech Daresbury Warrington WA4 4AD UK; l Clarendon Laboratory, University of Oxford Oxford OX1 3PU UK

## Abstract

Proton transfer reactions are central to chemical and biological systems, where quantum effects—such as tunneling, delocalization, and zero-point motion—critically influence reaction kinetics. Classical methods that capture these phenomena scale poorly with system size, limiting their applicability. Here, we extend and benchmark a quantum computing framework based on the Nuclear–Electronic Orbital formalism, treating the transferring proton quantum mechanically, to assess the feasibility of computing accurate energy barriers on current quantum devices. Using malonaldehyde as a prototypical system, we construct deep initial circuits *via* ADAPT-VQE combined with the frozen natural orbital approximation and apply adaptive approximate quantum compiling to balance circuit depth and fidelity. Transpiling these circuits for the ibm_pittsburgh device and simulating with realistic noise models, we compute barrier heights and delocalized proton densities along the reaction pathway. Circuit refinement and compression yield compact representations that preserve essential quantum features of the transfer process. Notably, our shallowest circuits (AQC-low) reproduce key qualitative features, such as proton density localization, and are near the frontier of feasibility for current hardware. In contrast, deeper circuits (AQC-high) retain higher fidelity to reference barrier height, reducing the error to 1.6 mHa (13%) while still yielding a 98% underestimation of the rate constant at 120 K.

## Abstract 2

Variational quantum circuits are benchmarked for their ability to reproduce proton-transfer barriers and nuclear densities relevant to reaction kinetics.

## Graphical abstract

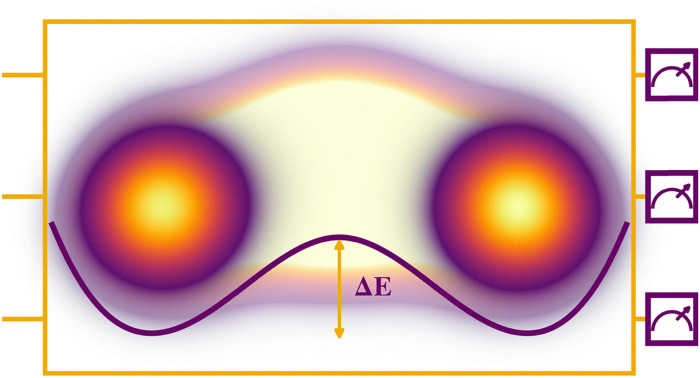

## I. Introduction

Proton transfer plays a crucial role in many physical and biochemical processes. For example, several enzymes, particularly those involved in metabolic pathways, use proton transfer as part of the catalytic cycle.^1^ Kinases,^2^ oxidoreductases,^3^ and dehydrogenases^4^ rely on the transfer of protons between the residues of the active site to catalyze reactions. Coupled proton and electron transfers are known to be important for drugs targeting mitochondrial function or redox-based mechanisms,^5^ and their rates can influence the overall reactivity and efficiency of drug candidates targeting redox enzymes. Similarly, proton transfer can be the rate-determining step in enantioselective reactions involving chiral substrates.^6^

Typically, the reaction rates *k*(*T*) of these proton transfer processes at a temperature *T* are related to the barrier height *E*_b_ by an Arrhenius law *k*(*T*) ∝ e^−*E*_b_/*RT*^, which assumes that the mobile nuclei behave classically.^7^ Given such exponential sensitivities, accurate prediction of the proton transfer energy barriers involved is critical for rate control and in the design of reactions that favor one pathway over another. However, a significant complexity arises because proton transfer often involves light nuclei, for which the classical approximation fails, leading to deviations from the classical Arrhenius behavior due to quantum mechanical phenomena such as zero-point energy, nuclear delocalization and tunneling. Consequently, reactions proceed at rates inconsistent with classical transition state theory. In these circumstances, treating the labile protons quantum mechanically is necessary to recover physically realistic models.

Several theoretical approaches have been developed over the past decades that can accurately describe proton transfer;^8,9^ however, they are mainly based on trajectories (quantum or classical) or on the quantization of vibrational modes. The nuclear–electronic orbital (NEO) approach,^10,11^ on the other hand, has emerged as a computationally efficient alternative for capturing nuclear quantum effects using localized basis functions. In the NEO method, both electrons and selected transferring protons are treated quantum mechanically with a molecular orbital theory approach. Therefore, the NEO framework offers an ideal computational platform for the simulation of proton transfer processes^12–16^ on a quantum computer. These simulations typically rely on multicomponent density functional theory (DFT) mean-field method^17–19^ either in the context of real-time NEO time-dependent density functional theory (RT–NEO–TDDFT)^12^ and RT–NEO–TDDFT Ehrenfest dynamics^13^ or multi-state NEO density functional theory (NEO–MSDFT).^20^ Due to the well-known limitations of currently available electron–electron^21^ and electron–proton functionals,^18,19^ these methods do not provide adequate accuracy to meet practical experimental demands^22^ (below 1 kcal mol^−1^), providing the impetus for the development of NEO methods of much higher accuracy.

On classical computers, the resources required for exact solutions of NEO-based models grow exponentially with system size.^11^ By contrast, advances in quantum computing technology and algorithm developments have opened a path for solving this problem nearly exactly on quantum devices in a more efficient way.^23–28^ As fault-tolerant quantum computing (FTQC) remains a prospect, algorithms tailored to address the challenges posed by noisy intermediate-scale quantum (NISQ)^29^ hardware are currently being developed.

In this work, we extend the recently developed NEO framework for modelling proton transfer reactions.^27^ First, we enhance the physical realism of the model by employing larger basis sets, the frozen natural orbitals (FNO) approach, and by accounting for scaffold relaxation effects. Second, we use the adaptive derivative-assembled pseudo-Trotter ansatz variational quantum eigensolver (ADAPT-VQE) algorithm for efficient wavefunction parametrization,^30^ followed by circuit compression using approximate quantum compiling (AQC),^31–33^ enabling applications on noisy intermediate-scale quantum (NISQ) hardware. Third, the resulting circuits, at varying levels of compression, are then used to simulate adiabatic proton transfer in the malonaldehyde molecule,^27^ with a focus on computing the transfer barrier in the presence of quantum nuclei.

## II. Theory

We investigate the quantum dynamics of proton transfer in malonaldehyde using a time-dependent quantum simulation framework. Rather than a static description, this approach captures the evolution of both electronic and nuclear degrees of freedom, guided along a well-defined, chemically relevant proton transfer pathway. The core of our approach is to solve the time-dependent Schrödinger equation (TDSE) for the coupled proton–electron system, leveraging the NEO framework. In Section II A we describe the TDSE without going into details regarding the actual molecular setup, which will be covered in Section II B.

### A. Coupled electron–proton dynamics

The dynamics of the system is governed by the TDSE1
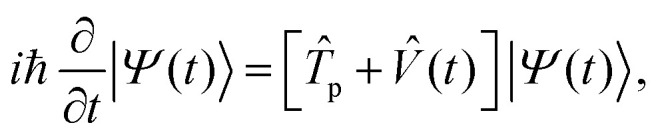
where *T̂*_p_ is the kinetic energy operator for the proton, and *V̂*(*t*) is a time-dependent potential incorporating both the proton's interaction with its environment and the coupling between protonic and electronic components. The explicit time dependence in *V̂*(*t*) arises from our protocol for steering the system Hamiltonian along the reaction pathway, as described in Section II B. The total time-dependent potential is given by*V̂*(*t*) = *V̂*_p_(*t*) + *V̂*_ep_(*t*) + *Ĥ*_e_(*t*)where *V̂*_p_(*t*) describes the time-dependent interaction of the proton with the classical nuclear scaffold, *V̂*_ep_(*t*) is the proton–electron interaction, and *Ĥ*_e_(*t*) defines the electronic Hamiltonian contribution. Specifically, the proton–classical–nuclear interaction is represented in second quantization as
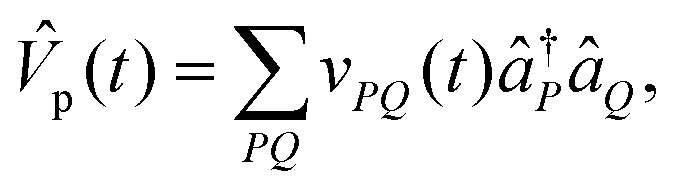
The coupling between protonic and electronic degrees of freedom is written as
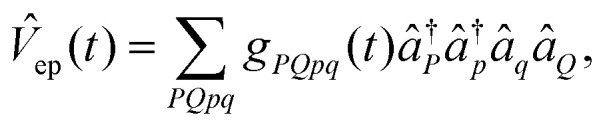
and the purely electronic part is given by
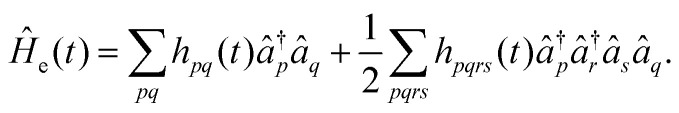
In this work, the lower case *p*, *q*, *r*, *s* indices denote general electronic spin orbitals, whereas the upper case indices denote protonic spin orbitals. The operators *â*^†^_*p*_ and *â*_*p*_ are creation and annihilation operators which satisfy the normal Fermionic anticommutation relations. The explicit time dependence of integrals *v*_*PQ*_(*t*), *g*_*PQpq*_(*t*), *h*_*pq*_(*t*), and *h*_*pqrs*_(*t*) arises from the fact that the underlying electronic molecular orbitals are defined with reference to a representative proton transfer pathway (see Section II B for more details). This prescribed evolution of the electronic orbitals steers the quantum dynamics of both the electronic and protonic degrees of freedom.

For the time-dependent wave function with protonic (nuclear) and electronic components |*Φ*^p^_*ν*_〉 and |*Φ*^e^_*μ*_〉 respectively, we write
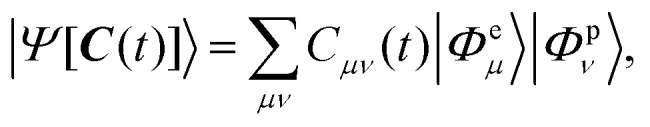
which inserted into eqn (1) leads to an equation of motion for the configuration interaction (CI) coefficients ***C***(*t*)2
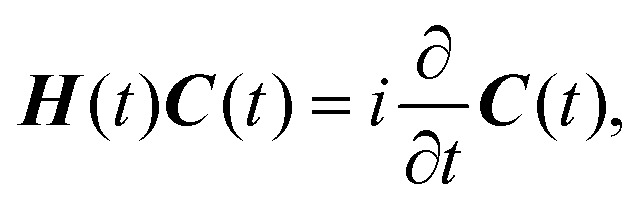
where the matrix elements of ***H***(*t*) are defined by

3 *H*_*κλ*,*μν*_(*t*) = 〈*Φ*^e^_*κ*_| 〈*Φ*^p^_*λ*_|*T̂*_p_ + *V̂*(*t*)|*Φ*^e^_*μ*_〉 |*Φ*^p^_*ν*_〉.

The rate of change of *V̂*(*t*) determines whether the system is in the adiabatic or non-adiabatic regime. Previously, Kovyrshin *et al.* (2023)^27^ explored dynamics through Suzuki decomposition in both cases. While highly accurate, this approach requires quantum circuits of substantial depth for time evolution—a limitation for near-term quantum hardware.

To address these computational challenges, we adapt our protocol for compatibility with variational quantum algorithms, specifically the variational quantum eigensolver (VQE),^34^ thus extending the application of VQE to simulation of dynamics in the adiabatic regime. Rather than simulating explicit real-time evolution according to eqn (2), we approximate the time-dependent energy and wavefunction variationally at each point along the reaction pathway; that is, at each time, the energy and ground state of the instantaneous proton–electron Hamiltonian are obtained by minimizing4
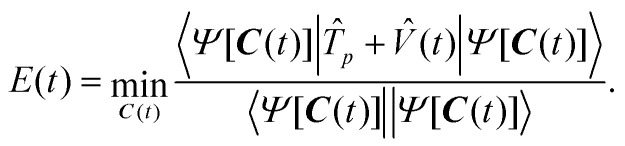


Hence eqn (2) is introduced for context, but the quantum dynamics are simulated by variational minimization rather than explicit numerical integration. Although limited to the adiabatic regime—*i.e.*, evolution under a slowly varying time-dependent Hamiltonian—this strategy allows for quantum simulation of proton transfer using compact variational ansätze of nearly constant size. This makes the problem tractable on near-term devices while still incorporating electron–proton coupling and capturing key quantum effects such as zero-point energy and proton delocalization.

### B. Malonaldehyde setup

The process of proton-coupled electron transfer (PCET) in malonaldehyde,^35^ a prototypical model for enantioselective isomerization, has been previously studied by Kovyrshin *et al.*^27^ in adiabatic and nonadiabatic limits. That study served as a proof-of-concept demonstration, which was limited to near-minimal electronic and nuclear basis sets. In this work, we focus on enhancing the physical realism of the model to obtain quantitatively accurate results comparable to experimental measurements. This is done by employing a larger basis set for electronic and protonic orbitals (see Appendix A), allowing scaffold relaxation,^35^ and leveraging the FNO technique.^30^ Next, we describe the procedures for generating the set of molecular orbitals needed to represent the system and for modelling the proton transfer through the adiabatic steering of the system Hamiltonian.

Similarly to, ref. ^27^, we describe the dynamics of proton transfer by first generating the structure of the molecule at three stationary points along a predefined reaction path. These describe the initial (Left), transition (Middle) and final (Right) states respectively. These structures and their corresponding molecular orbitals, which are needed to model the system, are obtained in three steps.

**Fig. 1:**
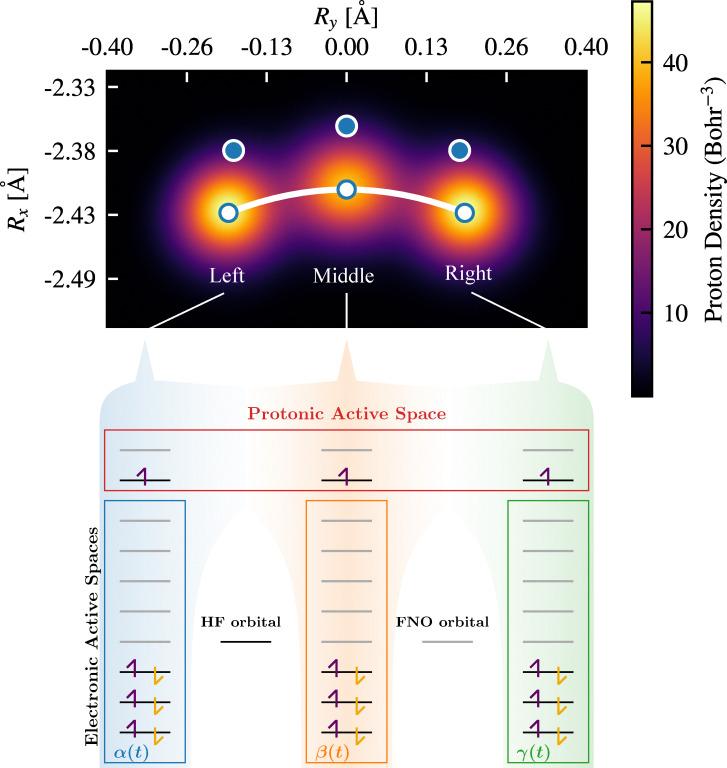
Proton density distribution for Left, Middle, and Right setups computed from the FNO–NEO–CASCI protonic one-particle density matrix. The proton density extends downward, and the classical nuclei lie above the plotted region. White points indicate the expectation values of the proton position operator for Left, Middle, and Right setups, while blue points denote the positions of the protonic orbital centers used in the basis set expansion. The overlaid white trajectory indicates the expectation value path of the proton position. Schematic illustration of the electronic and protonic active spaces used in the Left, Middle, and Right Hamiltonians. Gray lines indicate the FNO orbitals, and black lines represent the HF orbitals. For each setup, the occupation of the lowest energy product state (HF state) is shown.

First, the three possible positions for the H atom and the corresponding scaffolds are modelled within the Born–Oppenheimer approximation, treating all nuclei as classical point particles. This is done using second-order Møller–Plesset (MP2) theory, which yields the fully optimised structure under the *C*_s_ point-group symmetry (see Appendix A for basis set specifications). The corresponding geometries are provided in Table 4 in Appendix B. Thus, in contrast to ref. 27, our setup includes scaffold relaxation upon proton transfer. The resulting proton positions (see blue points in Fig. 1 and Table 5 in Appendix B) serve as a good initial guess for the subsequent step where we go beyond the Born–Oppenheimer approximation by treating the transferring proton quantum mechanically. Second, we perform one nuclear–electronic orbital Hartree–Fock (NEO-HF) calculation per scaffold, keeping the protonic orbitals centered at these positions. This step yields the occupied protonic and electronic molecular orbitals for the system. Third, to obtain more accurate virtual orbital space (*i.e.*, unoccupied orbitals), a higher accuracy method—NEO–MP2—is used to generate the corresponding NEO–FNO orbitals (see Section III B for more details).

The construction of the orbitals for the Right, Middle and Left configurations enables us to define a Hamiltonian which represents a changing external potential in which the proton under study evolves. In order to describe this situation, we construct the three Hamiltonian operators in second quantization based on the sets of protonic and electronic orbitals derived above. The protonic active space (AS) was chosen to contain two orbitals from Left, Middle, and Right setups, and is shared among the three Hamiltonians. Specifically, one occupied NEO-HF and one FNO orbital from each setup result in 6 protonic orbitals. As these orbitals originate from separate calculations, they are not orthogonal, and a Löwdin orthogonalization procedure^36^ was performed before inclusion of these to protonic (AS). While the same protonic AS was used for all Hamiltonians, the three different electronic AS were comprised of 3 NEO-HF and 5 FNO orbitals from Left, Middle, and Right configurations (the 16 lowest occupied orbitals were considered frozen). Thus, the protonic and electronic active spaces were doubled in size compared to ref. 27, and six electrons were considered in the active space instead of four. A schematic representation of the active spaces is shown in the lower part of Fig. 1.

First, we benchmark the combined effect of these methodological improvements by comparing active space configuration interaction (CASCI) energies computed for the Left and Middle Hamiltonians using our current approach, FNO–NEO–CASCI, with the results from Ref. 27, referred to as NEO–CASCI. In Table 1 we list the calculated energy barrier, Δ*E* = *E*_M_ − *E*_L/R_, for the two methods along with the experimental and semi-empirical values for comparison. We find that FNO–NEO–CASCI doubles the proton transfer barrier compared to ref. 27, yielding a value of 11.9 mHa. This value falls between the experimentally inferred estimate of approximately 13.5 mHa^37^ and the semi-empirical model-derived value of approximately 11.2 mHa.^38^ The former is based on a broad IR absorption near 2960 cm^−1^ tentatively attributed to motion along the proton transfer coordinate. Notably, the Born–Oppenheimer approximation systematically overestimates the barrier,^27,39^ yielding 16.4 mHa when applied in CASCI with the current basis set and structures (see Table 1).††Even though the Born–Oppenheimer CASCI barrier reported in ref. 39 lies close to the experimental value, this agreement reflects fortuitous error cancellation arising from basis-set incompleteness and insufficient correlation—a classic “Pauling point” in the sense of Löwdin.^40^ In addition, we observe an effect on proton–electron entanglement within the system. The entanglement entropy is calculated from the single-proton reduced density matrix, obtained by tracing out the electronic degrees of freedom, for the Left, Middle, and Right ground states. As shown in Table 1, a considerable increase in entanglement is observed for the Middle state, while Left and Right states show only minor improvements. This indicates that the new framework, FNO–NEO–CASCI, is better suited to capturing proton–electron entanglement compared to NEO–CASCI.^27^

**Table 1:** Proton-transfer energy barriers obtained with different methods. NEO–CASCI from ref. 27 (*) and FNO–NEO–CASCI reference values are reported together with corresponding entanglement entropy values for Left, Middle, and Right setups. For comparison, the Born–Oppenheimer CASCI (BO-CASCI) barrier is also included

Method	Δ*E* (mHa)	Entanglement entropy	
		Left/Right	Middle
NEO–CASCI	5.1	0.00200	0.0038
FNO–NEO–CASCI	11.9	0.00240	0.0066
Semi-empirical model^38^	11.2		
Experimental^37^	13.5		
BO-CASCI	16.4		

To assess the spatial delocalization arising from the quantum treatment of the proton, we analyze the protonic density and deviation from the classical reference position. Specifically, we evaluate the protonic densities and the expectation values of the proton position operator from a protonic one-particle density matrix constructed for Left, Middle, and Right FNO–NEO–CASCI states (see Fig. 1). The corresponding proton densities reveal a strongly delocalised character across all three setups, emphasizing the inadequacy of a purely classical treatment. The expectation values for the proton position (white points in Fig. 1 and Table 5) exhibit substantial displacement from the original centres of protonic basis functions—*i.e.*, the classical reference positions. These findings highlight the importance of quantum nuclear effects where proton motion plays a central role.

With the Left, Middle, and Right Hamiltonians prepared (see Appendix A for more details), we now define the time-dependent Hamiltonian for the system

5 *H*(*t*) = *α*(*t*)*H*_Left_ + *β*(*t*)*H*_Middle_ + *γ*(*t*)*H*_Right_.

This is then used in eqn (8) to adiabatically change the system from *H*_Left_ through *H*_Middle_ to *H*_Right_, by choosing the parameters *α*, *β* and *γ*, such that they define the adiabatic trajectory for proton transfer, as governed by eqn (2) and (4), where the Left, Middle, and Right energies can be considered as stationary points along this trajectory (see Section IV B for more details on selecting *α*, *β* and *γ* such that the adiabatic trajectory mimics this physical process).

### C. Transition rate constant

To highlight the importance of accurately estimating the reaction barrier, it is useful to demonstrate its effect on reaction kinetics. The temperature-dependent rate constant for proton transfer, incorporating quantum nuclear effects *via* the NEO method, can be computed using a transition state theory (TST) expression^7^6
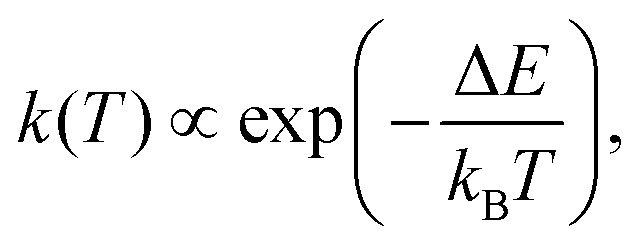
where *T* is the temperature and *k*_B_ is Boltzmann's constant. This formulation parallels the Eyring equation^7^ but uses an effective quantum barrier, Δ*E*, instead of Gibbs free energy of activation. The barrier incorporates proton zero-point energy and proton delocalization effects, making the expression particularly suitable for the light-particle transfer process. Although thermal and entropic contributions are omitted, this is a reasonable approximation given their relatively minor role in the short-timescale proton transfer process.

## III. Methods

The methods presented in this section detail the computational pipeline used to simulate quantum proton transfer dynamics in malonaldehyde. We begin with a schematic overview of our multi-stage workflow, which integrates accurate *ab initio* reference calculations, variational quantum algorithms, circuit compression, and error mitigation techniques. Subsequent subsections provide in-depth descriptions of the ADAPT-VQE method for quantum wavefunction preparation, the frozen natural orbital concept, the adaptive approximate quantum compiling (ADAPT-AQC) approach for circuit depth reduction, and the implementation of zero-noise extrapolation (ZNE) to address the effects of hardware noise in realistic quantum simulations.

### A. Pipeline overview

**Fig. 2:**
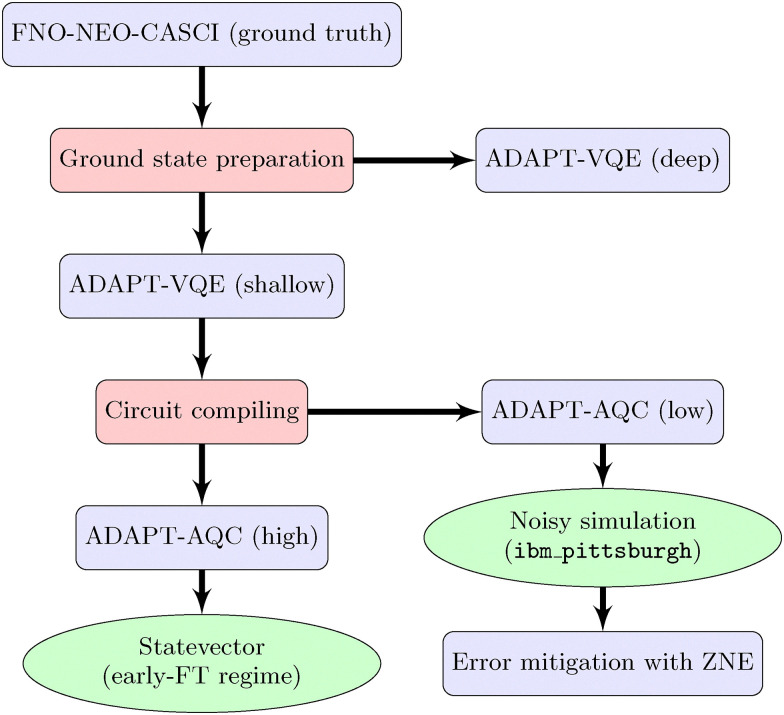
Schematic of our multi-stage proton-transfer simulation workflow. We begin with the high-precision FNO–NEO–CASCI reference (blue block) and perform ADAPT-VQE optimization (red block). By running the VQE driver to two distinct convergence thresholds, we generate both deep and shallow ADAPT-VQE circuits. These are then passed through adaptive approximate quantum compiling (ADAPT-AQC, red block), yielding high- and low-fidelity contracted circuits with significantly reduced two-qubit depth compared to ADAPT-VQE shallow. The ADAPT-AQC deep circuit is simulated in the noiseless regime using a state-vector simulator, while the ADAPT-AQC shallow circuit is executed on the noisy ibm_pittsburgh model combined with ZNE error mitigation.

The computational workflow for simulating quantum proton transfer dynamics in malonaldehyde is illustrated in Fig. 2. Our pipeline integrates high-level *ab initio* electronic structure theory with state-of-the-art quantum algorithms, ensuring both accuracy and practical feasibility for near-term quantum hardware. The steps are as follows.

First, we compute ground-state energies using the FNO–NEO–CASCI approach described in Section II B, which we refer to as CASCI in the following. This provides a reliable theoretical reference for benchmarking proton transfer barriers and quantum simulation outcomes.

Next, the ADAPT-VQE (see Section III B for details) is used to prepare quantum circuits representing the ground-state wavefunction for the time-dependent Hamiltonian at different times. This procedure is carried out at two distinct accuracy thresholds: an energy error of 10^−3^ Ha and a less conservative value of 10^−2^ Ha, relative to the CASCI reference energy. The selected convergence threshold affects the required circuit depth, balancing computational accuracy and quantum hardware feasibility. The resulting deep circuit, VQE-deep, will be used as a benchmark to represent the early fault-tolerant regime, whereas the shallow circuits, VQE-shallow, will be further compressed as described below. To ensure compatibility with near-term quantum devices, where circuit depth and gate error rates are critical constraints, we further compress the ADAPT-VQE circuits utilizing ADAPT-AQC.^41^ This procedure significantly reduces circuit depth at the cost of a further accuracy trade-off. The method provides control over the level of compression tailored to different hardware capabilities. In our specific case, we produce both high- and low-fidelity compiled circuits, denoted AQC-high and AQC-low.

The compiled circuits are subsequently simulated in two regimes: (1) the high-fidelity circuit is used in an ideal, noiseless statevector simulator (representative of the early fault-tolerant quantum era), and (2) the low-fidelity circuit is used under realistic noise models based on the ibm_pittsburgh Heron processor, which includes comprehensive device imperfections (see Appendix C for details). For the latter, we apply ZNE, an error mitigation protocol, to recover expectation values closer to the noise-free limit.

The following sections provide further details for the ADAPT-VQE, FNO, ADAPT-AQC, and ZNE techniques.

### B. ADAPT-VQE with NEO–FNO pool

At the core of our quantum simulation protocol is the ADAPT-VQE algorithm,^30^ applied to a pool of the NEO excitation operators spanning the space defined by the FNO approach. It is used to approximate the ground state wavefunction |*Ψ*[***C***(*t*)]〉 for each time-dependent Hamiltonian along the adiabatic trajectory. This method, detailed in ref. 30, delivers a wave function with a compact circuit and high accuracy; we briefly review it below. As an extension of its electronic counterpart,^42^ it bears similar features: the wavefunction ansatz is built iteratively by adding operators from a pool comprising single and double electronic, protonic, and mixed Fermionic excitations:*τ*^*μ*^ ∈ {*a*^*a*^_*i*_ − *a*^*i*^_*a*_, *a*^*A*^_*I*_ − *a*^*I*^_*A*_, *a*^*ab*^_*ij*_ − *a*^*ij*^_*ab*_, *a*^*AB*^_*IJ*_ − *a*^*IJ*^_*AB*_, *a*^*aA*^_*iI*_ − *a*^*iI*^_*aA*_},where *a*^*a*^_*i*_ = (*a*^*i*^_*a*_)^†^ = *a*^†^_*a*_*a*_*i*_, *a*^*A*^_*I*_ = (*a*^*I*^_*A*_)^†^ = *a*^†^_*A*_*a*_*I*_ are single electronic and protonic, respectively, excitation operators, and *a*^*ab*^_*ij*_ = (*a*^*ij*^_*ab*_)^†^ = *a*^†^_*a*_*a*^†^_*b*_*a*_*j*_*a*_*i*_, *a*^*AB*^_*IJ*_ = (*a*^*IJ*^_*AB*_)^†^ = *a*^†^_*A*_*a*^†^_*B*_*a*_*J*_*a*_*I*_, and *a*^*aA*^_*iI*_ = (*a*^*iI*^_*aA*_)^†^ = *a*^†^_*a*_*a*^†^_*A*_*a*_*i*_*a*_*I*_ are double electron–electron, proton–proton, and electron–proton excitation operators. Here, the lower-case *i*, *j*, *k*, *l*, …, *a*, *b*, *c*, *d*, … indices stand for occupied and virtual electronic spin orbitals, respectively. The protonic spin orbitals are defined analogously using upper-case indices. At each iteration, the operator that most effectively lowers the system energy is selected and added to the variational circuit together with its optimised parameter. After the *n*-th iteration, the ADAPT-VQE ansatz thus takes the form

7 |*Ψ*^(*n*)^_ADAPT-VQE_〉 = e^*θ*_*n*_*τ*^*n*^^…e^*θ*_2_*τ*^2^^e^*θ*_1_*τ*^1^^|0^e^0^p^〉

where (|0^e^0^p^〉 = |0^e^〉 ⊗ |0^p^〉) is the reference NEO-HF state composed from the electronic (|0^e^〉) and protonic (|0^p^〉) Slater determinants, and *θ*_*n*_ are the ansätze parameters. The ansatz grows until the convergence defined by a tolerance threshold is achieved. Further reduction in the circuit depth is achieved by selecting the operators from the qubit pool instead of the Fermionic pool. The qubit pool of operators is constructed by mapping the Fermionic operator pool to the qubit space and splitting the operators into individual strings.^43–45^

#### 1. Frozen natural orbitals approximation

To account for the missing dynamical correlation between quantum particles, we utilise the FNO approximation. The FNO approximation provides a means for systematic truncation of the unoccupied orbitals without sacrificing accuracy.^46^ As previously introduced in ref. 30, the electronic and protonic FNOs are defined as eigenvectors of a one-particle electronic density matrix*γ*^*b*^_*a*_ = 〈*Ψ*_NEO_|*a*^*b*^_*a*_|*Ψ*_NEO_〉and the protonic one-particle density matrix*γ*^*B*^_*A*_ = 〈*Ψ*_NEO_|*a*^*B*^_*A*_|*Ψ*_NEO_〉respectively, whereas their eigenvalues correspond to the occupation numbers. The FNOs with larger occupation numbers will contribute more to the total correlation energy, and those with a small occupation number will have insignificant contributions to the correlation energy and can therefore be discarded. Following this procedure, only the truncated set of unoccupied orbitals is used to define the active space and construct the pool of excitation operators in the ADAPT-VQE ansatz (eqn (7)), thereby reducing the number of qubits and gates required for the quantum computation. Here, the electronic and protonic density matrices are computed with the NEO first-order Møller–Plesset (NEO–MP2)^47,48^ wave function (|*Ψ*_NEO_〉) as detailed in Section II B. For more detailed expressions of these, we refer to eqn (S56) and (S58) of ref. 47, respectively.

### C. Circuit compression with approximate quantum compiling

The quantum circuits constructed *via* ADAPT-VQE, while compact relative to traditional approaches, may still exceed the limitation of current quantum hardware, particularly in two-qubit gate depth. To further reduce circuit depth, we apply ADAPT-AQC.^41,49^ In this variational approach, a parameterised circuit *V̂*(***θ***)|0〉 is optimised to approximate the target ADAPT-VQE state by minimizing the fidelity-based cost function

8 *C* = 1 − |〈*Ψ*_ADAPT-VQE_|*V̂*(***θ***)|0〉^⊗*n*^|^2^.

The variational ansatz is not assumed to have a fixed structure, but is instead built incrementally. At each increment, the algorithm adds a two-qubit unitary to the circuit, with the choice of qubits depending on the current state of optimization. For more details on the algorithm, we refer the reader to the literature.^33,41^ Notably, this method is itself inspired by ADAPT-VQE, and the combination of both methods demonstrates the complexity of our approach. Furthermore, AQC has been numerically demonstrated for up to 100 qubits and ADAPT-AQC up to 50 qubits by using tensor network simulations.^41,50^ This indicates the potential scalability of the techniques in this work for helping to simulate proton transfer at large scales. In particular, whilst ADAPT-AQC compression requires the ground state to be classically simulable, it can be used as a first step in simulating a classically intractable dynamical process. In this work, it is clear that such a capability would allow us to relax the adiabatic assumption of proton transfer and simulate the process in real time.

It should be noted that the fidelity cost function (eqn (8)) is evaluated classically using the matrix product state (MPS) representation: *V̂*^†^(***θ***)|*Ψ*_ADAPT-VQE_〉. Therefore, the target state for compilation, in this case |*Ψ*_ADAPT-VQE_〉, must be efficiently represented as an MPS. AQC is a classical method designed to construct shallow circuits which approximate MPSs to a desired level of accuracy. AQC may be applied to a classically-intractable state, |*ψ*〉, provided that a circuit which prepares the state is already known: |*ψ*〉 = *Û*|0〉. The circuit *Û* can always be factored into two parts: *Û* = *Û*_1_*Û*_0_, such that the initial part *Û*_0_ produces a state |*ψ*_0_〉 = *Û*_0_|0〉 which can be efficiently represented as an MPS. By using AQC to find a shallow approximation of |*ψ*_0_〉, denoted 
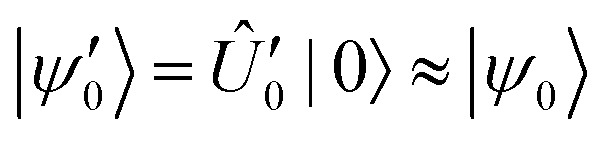
, a shallower approximation of the original state |*ψ*〉 may be obtained by 
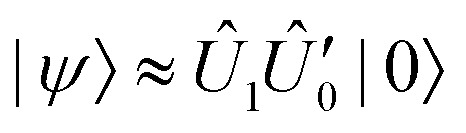
.

### D. Error mitigation using zero-noise extrapolation

The quantum computing NEO framework has been shown to produce accurate energy estimates in idealised, noise-free simulations.^27,30^ In this work, we aim to study the effects of hardware noise and assess the ability to recover the expectation values through error mitigation. This involves performing simulations that take into account sampling (shot) noise and a realistic device noise model derived from noise characterization protocols on the ibm_pittsburgh system.‡‡The ibm_pittsburgh quantum device contains 156 superconducting qubits on a Heron (3rd-generation) architecture. These qubits are connected *via* tunable-coupler technology in a heavy-hexagonal pattern. In this work, we apply the ZNE technique,^51,52^ which involves deliberately amplifying the noise in a quantum circuit—typically by repeating certain gates—and measuring the resulting expectation values at different noise levels. By fitting these noisy results to an analytical curve and extrapolating back to the zero-noise limit, ZNE provides an improved estimate of the expectation value that would be obtained on an ideal, noise-free device.

For noisy simulations, we use the device characterization data (see Appendix C), including qubit-specific *T*_1_, *T*_2_ coherence times, single- and two-qubit gate error rates, and readout errors, as determined from randomised benchmarking and echo protocols.^53,54^ The noise model selected corresponds to an error per layered gate (EPLG) of 0.001471 across 18 qubits, representative of device behavior during the study. The employed noise model includes (i) depolarizing channels derived by the 1- and 2-qubit gate error rates, followed by a thermal relaxation channel derived from the *T*_1_, *T*_2_ times and the corresponding gate lengths;^55^ (ii) bit-flip errors on the measurement outcomes derived from the readout error rates (see Appendix C).

Following the ZNE description in ref. 51, we first run the original circuit to obtain the unmitigated expectation value. Subsequently, copies of the circuit with folded gates to amplify the noise^56^ are run, where the gate-folding steps are performed locally on randomly selected gates, using the Mitiq noise-mitigation software package.^57^ The noise-amplified circuits, alongside the original circuit, are executed using the Qiskit Aer simulation library^58^ to compute noise-free and noisy expectation values. The resulting noisy expectation values are fit to a linear or quadratic function of the noise scaling factor *λ* ∈ [1, 4], and extrapolated back to *λ* → 0 to estimate the noise-free energies and barrier heights, as demonstrated in Fig. 5 and 6.

In this work, we adopted ZNE with 2-qubit gate unfolding as a standard and widely implemented benchmark approach for error mitigation. While other techniques—such as probabilistic error amplification (PEA)^59^ and probabilistic error cancellation (PEC)^60^—can offer improved accuracy when combined with noise suppression methods, they introduce significantly higher computational overhead (for both quantum and classical resources), particularly for Hamiltonians with thousands of terms requiring high-precision measurements. These advanced methods remain promising and will be the subject of future investigations, but were beyond the practical scope of the present study.

## IV. Results and discussion

### A. Barrier height evaluation

**Fig. 3:**
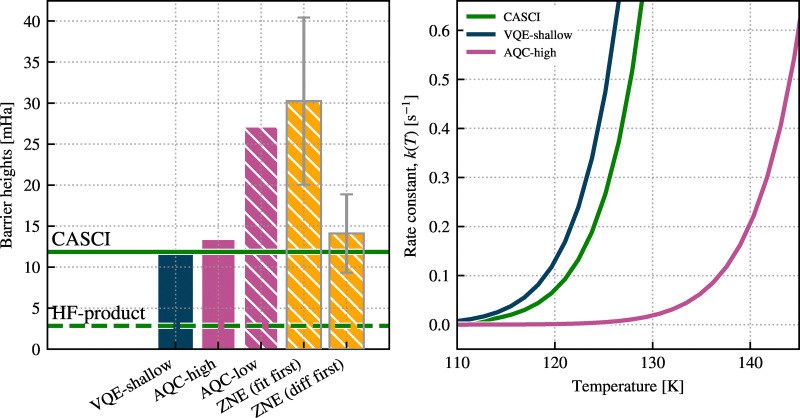
Left: Energy difference of the potential barrier (in mHa) for the VQE-shallow circuits, AQC-high and -low (noiseless, pink hatched), and the noisy AQC-low circuits with ZNE using two extrapolation methods (‘fit first’ and ‘diff first’, yellow hatched). The horizontal solid line indicates the CASCI result, and the dashed line the HF-product result (without correlations) for the energy barrier, as a guideline. The ZNE-based bars indicate the median value sampled from 100 randomised gate-folded circuits. The AQC-based methods overestimate the barrier height in the noiseless regime and, on average, in the presence of noise. Right: Proton transfer rate constants, *k*(*T*), as a function of temperature, *T*, computed using different methods based on a quantum-corrected TST expression of eqn (6). The green, blue, and pink lines indicate the CASCI, VQE-shallow, and AQC-high results, respectively. Energies estimated from AQC-low circuits would yield *k*(*T*) ≈ 0 in the temperature range shown and are omitted for clarity.

The computational pipeline introduced in Section III approximates the ground state wavefunction, with the aim of progressively reducing quantum circuit depth and thereby enabling execution on near-term devices. In Table 2 and Fig. 3 (left panel), we report the computed energies and barrier heights, Δ*E*, for proton transfer in malonaldehyde using various quantum and classical approaches, while the comparison of temperature dependence for the rate constants is shown in the right panel of Fig. 3. The CASCI result serves as a high-level quantum chemistry benchmark, while various quantum circuit-based methods demonstrate different trade-offs between circuit depth and fidelity. All quantum circuits presented in this work are transpiled to the ibm_pittsburgh device with Heron r3 architecture,^61^ and quantum resource requirements for their implementation are shown in Table 2.

**Table 2:** Summary of the parameters for the ADAPT-VQE and ADAPT-AQC circuits, along with energies (*E*) and proton transfer energy barriers (Δ*E*) computed *via* statevector simulation and classical methods. For each method, Δ*E* is defined as the difference between the middle and left absolute energies. Two-qubit gate depths and counts refer to circuits transpiled for IBM Heron processors. Circuit fidelities are given with respect to the FNO–NEO–CASCI wavefunction. To improve readability and focus on meaningful energy differences, a constant offset of 265 mHa was added to the reported absolute energies. All energy values are derived from noiseless statevector simulations, except for the ‘ZNE’ method, which includes noise

Method	Label	State	2Q-count	2Q-depth	Fidelity	*E* [mHa]	Δ*E* [mHa]
FNO–NEO–CASCI	CASCI	Left	—	—	—	−600.666	
		Middle	—	—	—	−588.809	11.857
HF-product	HF-product	Left	0	0	0.888	−552.090	
		Middle	0	0	0.936	−549.241	2.850
ADAPT-VQE (deep)	VQE-deep	Left	1844	1362	0.998	−599.268	
		Middle	940	662	0.999	−587.497	11.771
ADAPT-VQE (shallow)	VQE-shallow	Left	551	411	0.971	−591.237	
		Middle	271	211	0.976	−579.609	11.628
ADAPT-AQC (high)	AQC-high	Left	81	51	0.961	−578.785	
		Middle	405	90	0.967	−565.358	13.427
ADAPT-AQC (low)	AQC-low	Left	81	51	0.961	−578.785	
		Middle	85	12	0.953	−551.645	27.140
ADAPT-AQC (low + ZNE)	ZNE (fit first)	Left	—	—	—	−572 ± 7	
		Middle	—	—	—	−542 ± 7	30 ± 10
	ZNE (diff first)	Left, Middle	—	—	—	—	14 ± 5

The Hartree–Fock state significantly underestimates Δ*E* compared to CASCI reference. This highlights the critical role of correlation for accurate barrier evaluation and the need for quantum circuits balancing entanglement depth with hardware feasibility. For this, we apply two layers of approximations as discussed in Section III. First, employing ADAPT-VQE in a noiseless statevector simulation regime, we construct circuits labelled VQE-deep and VQE-shallow, yielding energies within 10^−3^ Ha and 10^−2^ Ha of the CASCI ground state, respectively (see Table 2). The resource requirements of VQE-deep circuits are too large for execution on currently available quantum processors, while they deliver outstanding accuracy for energy barrier and absolute energies. Although VQE-shallow produces circuits with relatively less overhead and delivers outstanding accuracy with respect to CASCI, their practical deployment remains unrealistic in the near term. Specifically, the two-qubit gate depth is the most important factor for reducing noise on current superconducting quantum computers, where decoherence over time is more significant than errors from imperfect execution of gates.

We further optimise the VQE-shallow circuits using ADAPT-AQC running with two settings. The first, ADAPT-AQC (high), is set to retain high fidelity relative to the target circuit at the cost of a deeper solution. The second, ADAPT-AQC (low), is set to prioritise reducing the entangling gate depth, at the cost of lower fidelity. Running the algorithm under both of these settings produces two sets of circuits we label AQC-high and AQC-low, as shown in Table 2.

The AQC-high circuits are able to reduce the two-qubit gate depth of the VQE-shallow circuits by 88% and 57% for the Left and Middle systems, respectively, while still maintaining fidelity 99% relative to the VQE-shallow target. Furthermore, evaluating the states produced by the AQC-high circuits yields a barrier height within approximately 13% (1.6 mHa) of the CASCI reference, but leads to a 98% underestimate in the rate constant at 120 K (Fig. 3, right panel). This discrepancy arises due to the exponential dependence of rate constants on barrier height. To quantify the sensitivity of the rate constant to barrier height errors, we note that an error δ*E* in the barrier leads to a fractional error in the rate constant δ*k*/*k* approximately given by δ*k*/*k* ≈ −δ*E*/*k*_B_*T*. At 120 K, this implies that the barrier must be accurate to within ≈0.08 mHa (≈2 meV) in order to keep the error in the corresponding rate constant below 20%. This sets a stringent requirement on quantum energy estimation algorithms to yield reliable kinetics in this temperature regime. Reaching this level of precision remains a key challenge—and benchmark—for emerging quantum algorithms applied to chemical reaction dynamics.

The AQC-low circuits have a minimum fidelity of 97% with respect to the VQE-shallow target. Whilst no improvement over the AQC-high could be achieved for the Left system, a solution for the Middle circuit is found with only 12 two-qubit gate depth. Thus, the AQC-low circuits have small enough resource requirements to be realised on currently available quantum hardware. However, the cost of the significant depth reduction of the ADAPT-AQC (low) protocol means the evaluated circuits overestimate the barrier by approximately 130%, resulting in an overwhelming suppression of the computed rate constant across the entire temperature range. This result underscores the importance of high-precision energy estimation when using quantum algorithms for chemical kinetics. Nevertheless, we accept these inaccuracies as a trade-off in order to evaluate the feasibility of using the NISQ devices, which remain limited by the depth of circuits they can reliably execute.

Our results show that noiseless simulations can recover the proton transfer energy barrier accurately with significant compression from ADAPT-AQC. Circuits produced by AQC-high significantly reduce the depth of entangling gates compared to VQE-shallow while maintaining good accuracy; however, they are still too deep to be executed reliably on noisy devices. Further compromising fidelity in favor of lower depth, the circuits produced by AQC-low offer a more realistic scenario for noisy quantum hardware, though they severely overestimate the barrier. Nonetheless, this may represent the only currently feasible option for application on a real device.

### B. Proton transfer dynamics

To simulate the proton transfer processes, we construct 7 Hamiltonians defined along the adiabatic trajectory by interpolating between Left, Middle, and Right as defined in eqn (5). We use the nomenclature LMR to label the resulting time-dependent Hamiltonians and corresponding states, where each digit reflects the weight of the respective Left, Middle, and Right setups. For example, the label 210 corresponds to the following Hamiltonian:
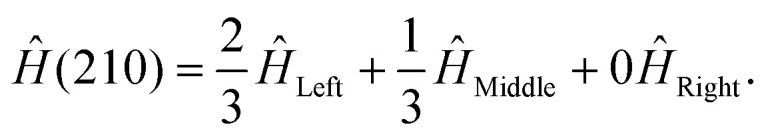
We then repeat the application of our pipeline in Fig. 2 on each of these Hamiltonians to produce a ground state for each intermediate point that can be run on current quantum hardware. Specifically, we use ADAPT-VQE (shallow), followed by ADAPT-AQC (low) to produce 7 circuits that approximate the ground state wavefunctions along the adiabatic proton transfer process for the time dependent Hamiltonian in eqn (5). Fig. 6 illustrates the full adiabatic proton-transfer pathway from the left to the right states. The plotted energies correspond to expectation values of the NEO Hamiltonian without the addition of the scalar constant arising from classical nuclear and frozen-core electron interactions. The hardware requirements for these circuits, along with the corresponding errors, are summarised in Table 3. One can observe shifts in energy across all states, which are not always systematic. In addition, asymmetries in the two-qubit gate depth of circuits are evident. Both effects originate from asymmetries in the ADAPT-VQE procedure, which initially uses the Hartree–Fock state of the Left setup (see Fig. 1) as a starting point for all steps along the adiabatic trajectory. These initial imbalances are inherited and further amplified by ADAPT-AQC compression.

**Table 3:** Summary of circuits contracted with ADAPT-AQC (low). Left and Middle are the same as in Table 2. The energy errors, in Hartree, are relative to the CASCI energy; we also report the two-qubit depth and the number of two-qubit gates in each synthesised circuit after transpiling for the ibm_pittsburgh heavy-hexagonal topology

State	Error (Ha)	2Q-depth	2Q-count
300 (Left)	0.022	51	81
210	0.043	12	87
120	0.037	12	86
030 (Middle)	0.037	12	85
021	0.039	12	87
012	0.044	20	67
003 (Right)	0.030	16	130

**Fig. 4:**
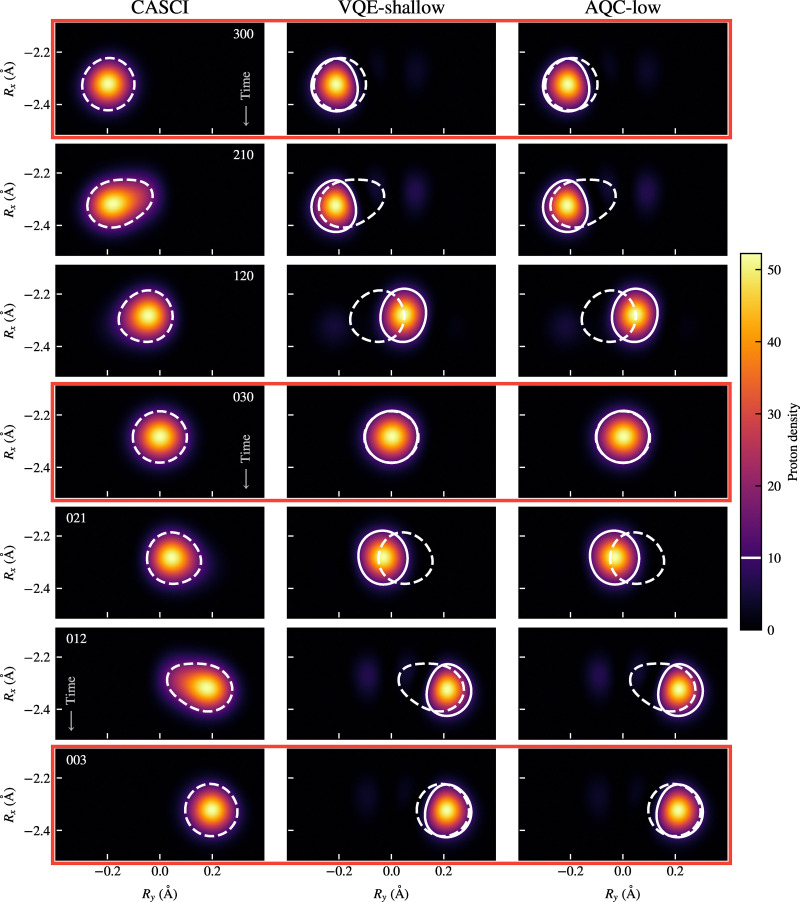
Proton density distributions obtained using the CASCI, VQE-shallow, and AQC-low methods. Each subplot corresponds to one computational method and shows the proton density for 7 positional configurations from Left (300) to Right (003) states. The Left, Middle, and Right densities (red boxes) most accurately reproduce the CASCI references, and their corresponding energies are used in barrier evaluation. The densities are evaluated on a 2D grid in the *XY* plane located at *R*_*z*_ = 0.0 Å.

For illustration purposes, we plot the proton densities along the proton transfer process in malonaldehyde. Fig. 4 compares these proton densities as computed using the increasing approximations of our pipeline: a high-accuracy CASCI reference (left), the VQE-shallow solution (middle), and the AQC-low solution (right).

The CASCI calculations show the exact nuclear delocalization at each geometry along the adiabatic transfer trajectory. The VQE-shallow solution qualitatively reproduces the key features of the nuclear density, particularly capturing the delocalization and correct spread of the proton wave packet at the Left, Middle, and Right states. However, at intermediate points along the reaction path, the VQE-shallow wave packets appear somewhat more localised than in the CASCI reference, suggesting that the variationally constructed ansatz, though compact, does not fully capture all the correlations needed for accurate delocalization at these states.

This limitation stems from the core approximations in ADAPT-VQE. The ansatz is constructed iteratively from a predefined operator pool, targeting ground-state energy minimization at each step. While this approach ensures efficiency and adaptivity, it can under-represent entanglement or spatial correlation effects that are not sufficiently prioritised by the energy gradient criterion used in operator selection, and these inaccuracies persist even in VQE-deep.

The right panel in Fig. 4 demonstrates how, despite its reduced depth, AQC-low solution almost exactly reproduces the key features of the VQE-shallow (middle panel) densities across the entire transfer pathway, including the subtle asymmetries and spatial localization patterns. This highlights the utility of AQC not only as a tool for circuit compression but also as a robust method for preserving chemically and physically meaningful wavefunction features, given an appropriate initial state.

### C. Hardware noise simulations

**Fig. 5:**
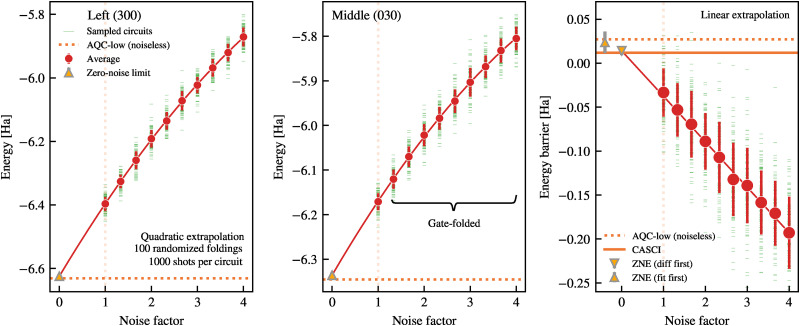
ZNE results for energy expectation values and proton transfer barrier heights across representative proton configurations in malonaldehyde, obtained from AQC-low circuits simulated under ibm_pittsburgh noise models. Ground-state energy expectation value (in Ha) as a function of noise amplification factor for Left (300, left panel) and Middle (030, central panel) proton configurations; the zero-noise limit (yellow markers) is obtained by extrapolation using a quadratic fit. The corresponding barrier energy, computed as the difference of the extrapolated intercepts, is indicated in the right panel as ‘fit first’ (upward-pointing yellow marker). On the right, we also show the barrier height measurements computed by the mean difference in raw energy between the Middle and Left states for each noise amplification factor (red markers). The zero-noise limit (downward-pointing yellow marker, ‘difference first’) is computed *via* a linear fit to the data (red line). Both ‘fit first’ and ‘difference first’ results are obtained by fitting the mean expectation values; the error bars are the variance in the correlation matrix element corresponding to the fitted intercept. In all panels, we indicate the noiseless energy value and barrier height from the AQC-low circuits (dotted lines); in the right panel, we indicate the CASCI reference energy barrier (solid horizontal line).

**Fig. 6:**
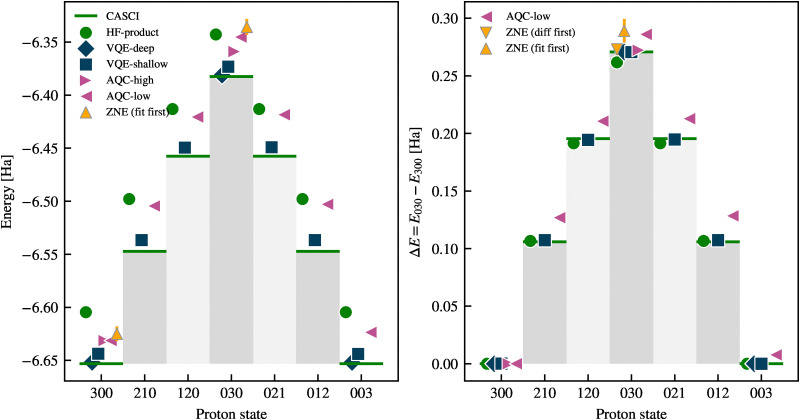
Energy profiles and relative barrier heights for the Left, Middle, Right and intermediate states of proton transfer in malonaldehyde computed along the adiabatic trajectory, comparing the following simulation methods: CASCI (green lines), HF-product (green circles), VQE-deep (blue diamonds), VQE-shallow (blue squares), AQC-high (purple right-pointing triangles), AQC-low (purple left-pointing triangles), ZNE fit-first (yellow upward-pointing triangles). Left: Absolute ground-state energies (in Ha) as a function of discrete proton configurations (denoted by the three-digit labels 300 → 210 → 120 → 030 → 021 → 012 → 003). Right: Energy barriers relative to the Left state energy, Δ*E* = *E*_state_ − *E*_300_ (in Ha). The circuits compiled with AQC-high achieve excellent agreement with the VQE and CASCI values.

In the following, we present results from the noisy simulations of the Left and Middle circuits, transpiled for the ibm_pittsburgh backend and executed with ZNE error mitigation. The ground-state energies for the Left (300) and Middle (030) configurations obtained with the AQC-low circuits under the ibm_pittsburgh noise model (see Table 6) are evaluated for noise amplification factor *λ* ∈ [1, 4]. For each value of *λ*, we constructed 100 distinct circuits with randomised folding of the 2-qubit gates. Each noise-scaled circuit is sampled 1000 times, and the noisy expectation values are then fitted to a quadratic function (see Appendix D for a discussion about model selection). The difference between the estimated intercepts for the Left and Middle configurations is used as an estimate for the ZNE energy. We refer to this as the ‘fit first’ method. The error bars in the ZNE energy estimates are given by standard error of the corresponding intercepts, which are added in quadrature to yield the 1*σ* uncertainty of the ‘fit first’ barrier energy. In Fig. 5 we plot the measured energies for the *H*_030_ and *H*_300_ along with the fitted lines that are used to estimate the ZNE energy of the barrier. Notably, the ZNE correction applied to the noisy simulations recovers the absolute energies for Left and Middle states within 6 mHa and 9 mHa respectively. While the absolute energy for Middle is slightly above HF results, we note that the noise simulations of the Left are able to capture some of the correlation energy (see Table 2 and Fig. 6). However, the barrier computed from these zero-noise limit (‘fit first’, yellow upward triangles in Fig. 5) closely approaches the ideal, noiseless circuit energies (dotted lines in the left panel of Fig. 5), *i.e.*, within 3 mHa on average. While this agreement may be partly coincidental—given that ZNE performs comparatively poorly for absolute energies—it suggests that the noise-scaling fit may better capture energy differences than absolute values.

Motivated by the above observation, we explore an alternative approach in which the energy difference between Middle and Left is first computed at each *λ*, and then the difference is extrapolated to *λ* = 0. We refer to this method as ‘difference first’. In the right panel of Fig. 5 we plot this energy difference along with a fitted, linear function (see Appendix D for a discussion about model selection). The ‘difference first’ method yields a barrier height of 14 ± 5 mHa (yellow, downward triangles in Fig. 6), which is lower than the ‘fit first’ result (30 ± 10 mHa). The uncertainty is also smaller (which is confirmed using non-parametric bootstrap estimation, see Appendix D for details), placing the noiseless AQC-low results 13 mHa away from the so-obtained zero-noise limit. Further investigation is needed to determine whether this effect is systematic or accidental.

These results suggest that, despite aggressive circuit compression the proposed framework remains too resource-intensive for near-term devices and will likely only become practical in the FTQC regime. Preliminary hardware experiments were also carried out on the ibm_pittsburgh device under conditions that differed from the simulated setups. Due to limited computational resources and the approximate nature of the noise model, these runs should be interpreted with caution. The results are therefore included in Section E of the Appendix for completeness rather than for quantitative comparison.

## V. Conclusions

This work establishes a practical framework for simulating proton transfer with quantum resources that are compatible with both near-term and future quantum computing architectures. By combining nuclear–electronic orbital methods with adaptive quantum circuit construction (ADAPT-VQE and ADAPT-AQC), we demonstrate that it is possible to approximate the proton transfer barrier in malonaldehyde with high accuracy while significantly reducing quantum circuit depth. Notably, our shallowest circuits, AQC-low, reproduce key qualitative features of the proton-transfer process, such as proton density localization, thereby positioning them near the frontier of feasibility for current hardware. By contrast, the deeper circuits, AQC-high, retain higher fidelity to the reference transfer barrier, with an error of 1.6 mHa, and point the way toward early fault-tolerant implementations. While the proposed approach reaches the frontier of what is currently achievable on NISQ hardware, reliable results remain premature even with noise mitigation and classically pre-optimized circuits. Nevertheless, the methodology offers a route toward constructing compact circuit initial states for proton-transfer kinetics within trotterized quantum time evolution^27^ on early fault-tolerant quantum devices.

Beyond malonaldehyde, this methodology offers a modular pipeline for tackling a broader class of proton-coupled electron transfer problems, where the interplay of light nuclei and electronic structure challenges classical methods. As quantum hardware continues to improve, the techniques presented here offer a path toward achieving chemical accuracy in simulating proton dynamics, representing a promising early application for the first generation of fault-tolerant quantum computers.

## Author contributions

Author contributions are listed according the CRediT (contributor roles taxonomy) classification; funding acquisition: S. M., J. C., A. B.; conceptualization: A. K., L. T., A. B.; methodology: A. K., L. T., E. A., A. B., S. B., D. M., A. N., F. P.; software: A. K., L. T., E. A., S. B., D. M., A. N., A. M.; investigation: A. K., L. T., D. M., E. A., A. N., A. M.; writing – original draft: A. K., L. T., S. B., D. M., E. A., F. P.; writing – review & editing: all authors.

## Conflicts of interest

The authors declare no conflict of interest.

## Supplementary Material

CP-028-D5CP04097C-s001

CP-028-D5CP04097C-s002

## Figures and Tables

**Fig. 7:**
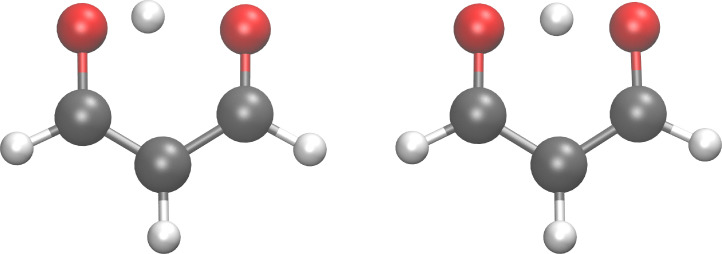
Optimized malonaldehyde structures corresponding to the Left and Middle setups.

**Fig. 8:**
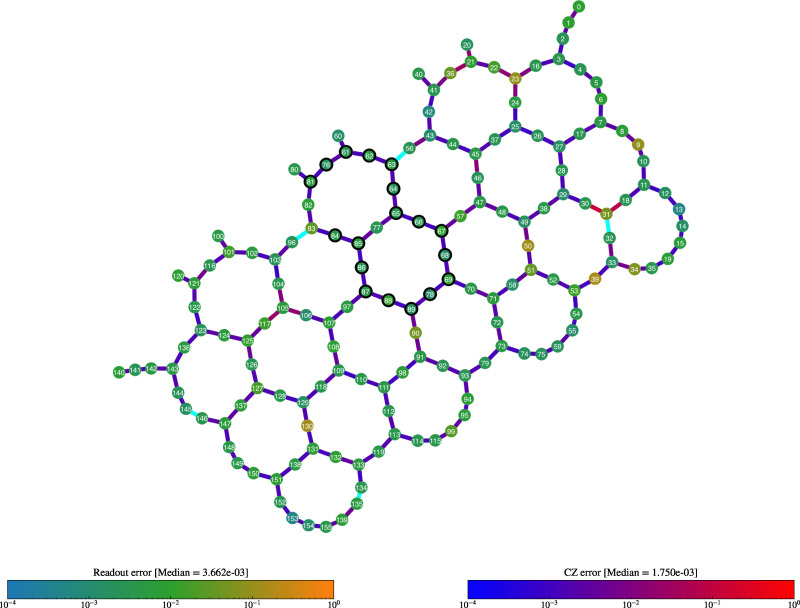
Error map of the ibm_pittsburgh processor captured for the calibration data recorded on 10 August 2025 at 18:59:40 UTC. Node colour represents the readout error of the physical qubits, and the edge colour represents the CZ error. Calibration data for two connections were not available (denoted in cyan). The physical qubits that correspond to a representative transpiled AQC-low circuit for the 030 state with a noise scale factor of 1 are indicated by black circles.

**Fig. 9:**
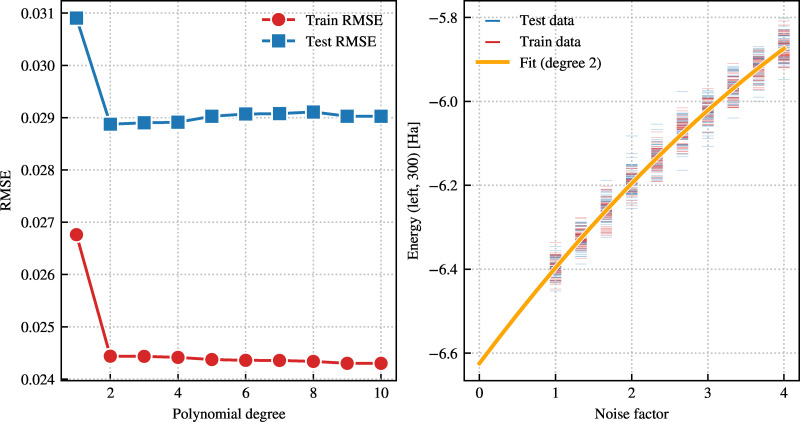
RMSE as a function of polynomial degree (left panel) and optimal fit to data (right panel) for the energies of the Left system *H*_300_.

**Fig. 10:**
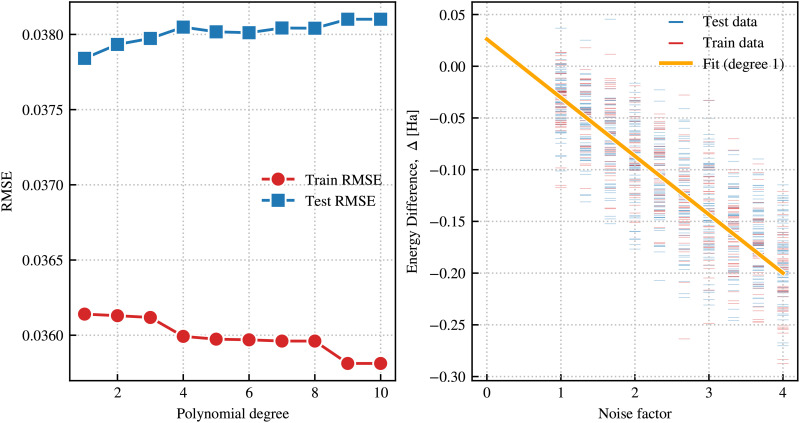
RMSE as a function of polynomial degree (left panel) and optimal fit to data (right panel) for the difference in energies between the Middle and Left systems *Δ* = *E*_030_ − *E*_300_.

**Fig. 11:**
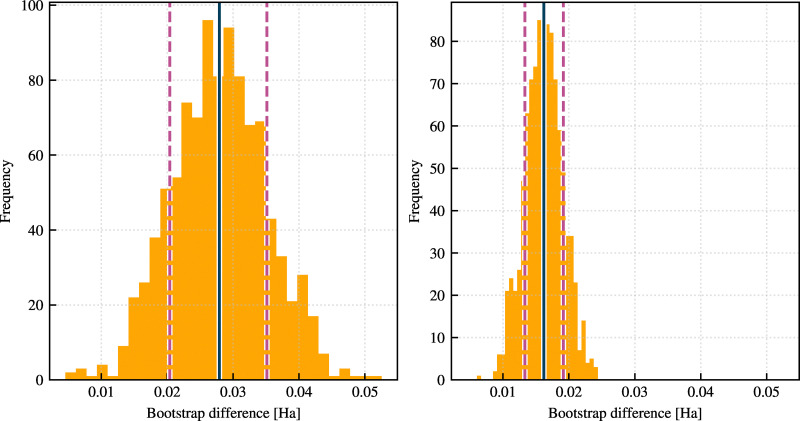
Empirical bootstrap distributions for the intercept for the ‘fit first’ (left panel) and ‘diff first’ (right panel) extrapolation methods. The solid line shows the median and dashed lines indicate the 15th and 85th percentiles, respectively.

**Fig. 12:**
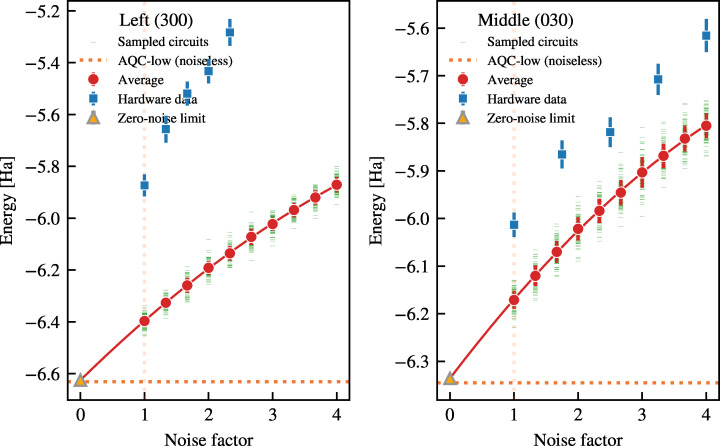
ZNE results of hardware experiments for the Left and Middle states of the AQC-low method, performed on ibm_pittsburgh device. The blue markers denote the hardware results, and noisy simulation results of Fig. 5 are included for reference.

## Data Availability

The data supporting this article have been included as part of the supplementary information (SI). Supplementary information is available. The Supplementary Information contains a video illustrating adiabatic proton-transfer dynamics and associated diagnostics within the NEO framework. See DOI: https://doi.org/10.1039/d5cp04097c. Data and code associated with this article is available as a public repository at https://github.com/stfc/quantum-neo-dynamics with DOI: https://doi.org/10.5281/zenodo.15924624. The repository contains (1) Hamiltonians and circuits used in this work, (2) demonstrative scripts to perform the simulations, and (3) the data generated from the simulations. Details on the basis sets, molecular coordinates, quantum device characteristics, and statistical analyses supporting this work are provided in the appendices of the article.

## Appendices

### Appendices

#### Appendix A: basis set setups and Hamiltonian constructions

The protonic orbitals are constructed using the PB-type 4s3p2d2f (PB4-F2)^69^ basis set, which is significantly larger than the split-valence double-*ζ* nuclear basis set composed of 2 uncontracted Cartesian *S* functions (DZSNB)^70^ used in ref. 27. For the electronic orbitals centered at positions of proton transfer, we use the ghost basis functions from the correlation-consistent polarised valence five-*ζ* (cc-pV5Z)^71^ basis set instead of split-valence double-*ζ* Gaussian basis set in 6-31 contraction scheme (6-31G). The single-*ζ* (minimal) basis set contracted from 6 Gaussian primitives (STO-6G),^72^ 6-31G,^73^ and correlation-consistent polarised valence double-*ζ* (cc-pVDZ)^71^ basis sets are used for the peripheral hydrogen, carbon, and oxygen atoms, respectively. This choice of basis sets improved the physical accuracy of the orbitals involved in PCET, leading to a more realistic representation of the underlying physics, while maintaining the total number of orbitals tractable.

The molecular integrals were generated using the GAMESS-US package, employing the *C*_s_ point-group symmetry. The frozen natural orbitals (FNOs) were constructed following the procedures described in ref. 47. The resulting one- and two-electron integrals were then mapped to a qubit Hamiltonian using the Jordan–Wigner transformation, yielding a 22-qubit representation. All present *Z*_2_ symmetries of the system were identified by applying the procedure introduced by Bravyi *et al.*^74^ to the Hamiltonian. This approach applies a Clifford transformation to the qubit Hamiltonian, allowing certain qubits to be treated classically and subsequently tapered off, thereby reducing the system to an 18-qubit Hamiltonian used in the simulations.

#### Appendix B: Cartesian coordinates and proton positions for malonaldehyde setups

Table 4 reports the full set of Cartesian coordinates (in Å) for the nine-atom malonaldehyde system in three representative proton-transfer configurations: the Left, Middle, and Right setups. Each configuration involves two oxygen atoms, three carbon atoms, and four hydrogen atoms (one of which participates directly in the intramolecular hydrogen bond). The molecular origin is placed at the central carbon atom, and all atomic positions are given relative to this point. The Left and Right geometries correspond to the proton localised on each oxygen, whereas the Middle geometry represents the proton symmetrically shared between the two oxygens. The structures corresponding to the Left and Middle setups are shown in Fig. 7.

**Table 4:** Cartesian coordinates (in Å) for all nine atoms in the Left, Middle, and Right malonaldehyde setups

	*x*	*y*	*z*
Left setup			
O	−2.01516150	1.25951633	0.00000000
O	−2.05554525	−1.16937003	0.00000000
C	−0.74721061	1.23462704	0.00000000
C	−0.72001126	−1.17773733	0.00000000
C	0.00000000	0.00000000	0.00000000
H	1.08331158	0.00000000	0.00000000
H	−0.20901142	2.19223326	0.00000000
H	−0.26674493	−2.16891499	0.00000000
H	−2.22176148	−0.18711600	0.00000000
Middle setup			
O	−2.04864177	1.15865439	0.00000000
O	−2.04864177	−1.15865439	0.00000000
C	−0.74990078	1.19199519	0.00000000
C	−0.74990078	−1.19199519	0.00000000
C	0.00000000	0.00000000	0.00000000
H	1.08244928	0.00000000	0.00000000
H	−0.27965698	2.17995088	0.00000000
H	−0.27965698	−2.17995088	0.00000000
H	−2.18129112	0.00000000	0.00000000
Right setup			
O	−2.05554525	1.16937003	0.00000000
O	−2.01516150	−1.25951633	0.00000000
C	−0.72001126	1.17773733	0.00000000
C	−0.74721061	−1.23462704	0.00000000
C	0.00000000	0.00000000	0.00000000
H	1.08331158	0.00000000	0.00000000
H	−0.26674493	2.16891499	0.00000000
H	−0.20901142	−2.19223326	0.00000000
H	−2.22176148	0.18711600	0.00000000

**Table 5:** Expectation values of the proton position operator (in Å), obtained from various ansätze. “Orb. center” referes to the position of the protonic orbital center from MP2 calculation

Method	*x*	*y*	*z*
Left setup—300			
Orb. center	−2.22176148	−0.18711600	0.00000000
CASCI	−2.32501550	−0.19558330	0.00000000
ADAPT	−2.32567623	−0.19555274	0.00000000
AQC	−2.32567623	−0.19555274	0.00000000
Middle setup—030			
Orb. center	−2.18129112	0.00000000	0.00000000
CASCI	−2.28629872	0.00000000	0.00000000
ADAPT	−2.28548356	0.00007774	0.00000000
AQC	−2.28548356	0.00007774	0.00000000

#### Appendix C: characteristics of IBM quantum devices

The ibm_pittsburgh device is a superconducting quantum processor built on IBM's third-generation Heron architecture, implementing 156 fixed-frequency transmon qubits arranged in a heavy-hexagonal lattice topology. This design minimises qubit crosstalk and enables efficient connectivity through tunable coupler technology.^54,75^ Each qubit is characterised by individual relaxation (*T*_1_), dephasing (*T*_2_), single- and two-qubit gate fidelities, and readout error rates, which are determined *via* standard quantum benchmarking protocols, including randomised benchmarking and Hahn echo sequences.^54,76^ Device performance is monitored using the error per layered gate (EPLG) metric,^77^ which quantifies the cumulative noise contribution across layers of a quantum circuit. A summary of noise models sampled is shown in Table 6. For this work, we selected a representative noise model based on calibration data recorded on August, 10th 2025 at 18:59:40 UTC, which yielded the lowest 18-qubit EPLG of 0.001471 across the sampled noise models. The corresponding analytic noise model incorporates depolarizing gate errors, thermal relaxation channels, and qubit-specific readout errors as captured by IBM's Qiskit Aer simulator. The qubit error map of the chosen noise model is shown in Fig. 8. While these characteristics represent the current state-of-the-art in superconducting quantum hardware, they continue to limit the accuracy of quantum simulations requiring high energy resolution, such as those involved in proton transfer dynamics.

**Table 6:** Date-time stamps for the noise models, and error per layered gate (EPLG) for 18 qubits. The noise model selected for simulations is highlighted

Timestamp (UTC)	EPLG 18 qubit error
2025-06-27 22:26:13 + 00:00	0.001620
2025-06-28 11:11:03 + 00:00	0.001779
2025-06-29 14:13:59 + 00:00	0.001653
2025-06-30 15:42:31 + 00:00	0.001675
2025-07-01 17:20:35 + 00:00	0.001671
2025-07-04 01:49:06 + 00:00	0.001664
2025-07-05 03:12:27 + 00:00	0.001545
2025-07-10 12:10:28 + 00:00	0.001640
2025-07-12 15:06:02 + 00:00	0.001679
2025-07-13 16:02:19 + 00:00	0.001669
2025-07-14 20:01:47 + 00:00	0.001776
2025-07-17 04:37:45 + 00:00	0.001510
2025-07-20 10:58:33 + 00:00	0.001689
2025-08-05 12:50:53 + 00:00	0.001654
**2025-08-10 18:59:40 + 00:00**	**0.001471**
2025-08-12 13:59:19 + 00:00	0.001778
2025-08-17 03:00:45 + 00:00	0.001726
2025-08-24 06:54:56 + 00:00	0.001724
2025-09-02 07:58:00 + 00:00	0.001786
2025-09-05 15:31:25 + 00:00	0.001691

#### Appendix D: zero-noise extrapolation with bootstrap resampling

To identify the most appropriate model describing the relationship between the noise factor and the measured energy output from the quantum device, polynomial regression models of varying degrees were evaluated. Model performance was quantified using the root mean squared error (RMSE), calculated separately for a hold out test set where the optimal polynomial degree was determined as the one minimizing this quantity, thereby achieving a balance between model complexity and generalizability while avoiding bias towards the intercept at *λ* = 0. In Fig. 9, the RMSE is shown as a function of polynomial degree for the measured energies of the Left system (*H*_300_). As illustrated, the lowest RMSE is attained for a polynomial degree of 2. In Fig. 10, the same procedure is applied to the difference in energies between the Middle and Left systems Δ*E* = *E*_030_ − *E*_300_ which is used to compute the ‘difference first’ barrier height shown in Table 2. In this case, the optimal polynomial degree is found to be 1. In both figures, model fits corresponding to the selected optimal polynomial degrees are presented alongside the observed data to enable qualitative assessment of model performance.

Given the optimal polynomial degree, bootstrap resampling was employed to estimate the variability and confidence intervals for the intercept parameter of the regression model corresponding to the error-mitigated estimate of the barrier height. To maintain the experimental structure, each bootstrap iteration consisted of resampling, with replacement, the observed data for each unique value of *λ*, thereby ensuring that the number of replicates per noise setting was preserved. For every resampled dataset, the polynomial regression model, using the previously determined optimal degree, was refitted, and the intercept coefficient was recorded. In Fig. 11, the empirical distributions for the ‘fit first’ and ‘diff first’ methods are presented. The ‘fit first’ distribution is derived by generating bootstrap samples of the intercepts for the Left and Middle systems, respectively, and taking their difference, whereas the ‘diff first’ distribution is derived by generating bootstrap samples of Δ*E* directly. The empirical distribution of the intercept values across all bootstrap replicates was subsequently used to quantify the uncertainty in this parameter. To capture the interval corresponding to ±1*σ*, the 15th and 85th percentiles of this distribution were used, while the median value served as a robust point estimate for the intercept. These results are summarized in Table 7 for both extrapolation methods. Excellent agreement is observed when comparing these bootstrap-based values with those obtained from estimation of the standard error of the intercept, as described in Section III D and presented in Table 2.

**Table 7:** Summary of ZNE extrapolation results using bootstrap estimation. All values are in mHa

Extrapolation method	Median	15th percentile	85th percentile
Fit first	24	15	32
Diff first	17	14	21

#### Appendix E: hardware experiments

To evaluate the performance of our circuits on quantum hardware, we performed ZNE on Left and Middle circuits of the AQC-low system on ibm_pittsburgh device.^78^ Hamiltonian of the Left state contains 5229 Pauli terms which can be grouped to 910 groups that preserve pairwise commutativity. As such, we performed ZNE for five noise amplification factors *λ* ∈ {1.0, 1.33, 1.66, 2.0, 2.33}. Each noise factor was sampled 2000 times to keep inline with the job execution limits.^79^ We utilised measurement error mitigation and Pauli twirling. For twirling, 2-qubit Clifford gate twirling was enabled and the number of randomisations and shots per randomisation were determined automatically as 32 and 64 respectively.^80^ Hamiltonian of the Middle state contains 567 commutative groups, which afforded us reach larger noise amplification factors *λ* ∈ {1.0, 1.75, 2.5, 3.25, 4.0} as well as increase the shot budget to 3500 per noise factor. Here too, we utilised measurement mitigation and twirling, and the number of randomisations was set to 300. Pauli twirling can improve the result quality of a hardware experiment as it turns coherent errors that accumulate quadratically into incoherent, statistical Pauli noise that accumulates linearly.

Results of the hardware experiments are shown in Fig. 12. As can be seen in Fig. 12, there appears to be an offset between the hardware results and the results from simulations subject to noise. While the Left state exhibits a large (>0.5 Ha) offset of energy for the unfolded expectation value, we note that this offset grows with the noise amplification factor as expected from gate-folding ZNE. The Middle state has a smaller offset around 0.2 Ha for the gate-unfolded case, however it shows a spurious constant offset across all noise factors. The offset between these two results may come from the simplified noise model used in simulations or from drift in the device noise profile since the time of calibration, at which the noise model was generated.

#### Appendix F: details of classical circuit compression

All ADAPT-VQE simulations were performed using a noise-free statevector simulator based on the full matrix representation of the quantum circuits, which eliminates the need for measurement-based sampling. The optimization of variational parameters was carried out using the L-BFGS-B algorithm similar to ref. 30. The ADAPT-AQC settings used for classical circuit compression are as follows:

**Table:**

Setting	Value
Optimiser	Rotosolve and rotoselect
Rotosolve frequency	1
Rotoselect tolerance	1 × 10^−5^
Rotosolve tolerance	1 × 10^−3^
Coupling map	Nearest-neighbour
Layer ansatz	U4

Further details about what these settings mean and the algorithm itself can be found in ref. 41. Rotosolve and rotoselect were developed in ref. 81. The U4 ansatz is based on a general two-qubit unitary as decomposed in ref. 82 such that each of the 15 single-qubit unitaries are parameterized.
