# Supplementary material for: Approximate quantum circuit compilation for proton-transfer kinetics on quantum processors
Source: Phys Chem Chem Phys. 2026 Jan 14;28(4):3035–54. doi: 10.1039/d5cp04097c (PMC12802553; doi:10.1039/d5cp04097c)
Supplement: CP-028-D5CP04097C-s002 [file CP-028-D5CP04097C-s002.pdf]

Supplementary Information (SI) for Physical Chemistry Chemical Physics.
This journal is © the Owner Societies 2025

Supplementary Information

Movie S1. Adiabatic Proton-Transfer Dynamics in Malonaldehyde

This video presents the reference adiabatic proton-transfer dynamics obtained by integrating the time-dependent Schrödinger equation using a fourth-order Runge–Kutta scheme within the NEO framework.
The **bottom panel** visualizes the **time-resolved proton density**, showing how the quantum proton delocalises and shifts between the Left and Right configurations during the simulated evolution.

The **top panel** contains three diagnostic plots evaluated at each simulation frame:

1. **Left plot:** Proton–electron **entanglement entropy**, indicating how nuclear–electronic correlation changes over the course of the adiabatic evolution.
2. **Middle plot: Instantaneous energy** of the evolving state with respect to the **Left (blue)**, **Middle (orange)**, and **Right (green)** Hamiltonians.
3. **Right plot: State fidelity** relative to the **Left (blue)**, **Middle (orange)**, and **Right (green)** ground states, showing how the evolving wavefunction aligns with each stationary reference state.

Together, these elements provide a detailed picture of proton delocalisation, energetic progression, and wavefunction character throughout the adiabatic proton-transfer process.
